# Experiences of support by unsuppressed adolescents living with HIV and their caregivers in Windhoek, Namibia: a qualitative study

**DOI:** 10.3389/fpubh.2024.1380027

**Published:** 2024-06-13

**Authors:** Farai K. Munyayi, Brian van Wyk

**Affiliations:** School of Public Health, University of the Western Cape, Cape Town, South Africa

**Keywords:** adolescents, HIV, viral suppression, antiretroviral therapy, fast-track city

## Abstract

**Background:**

Adolescents living with HIV (ALHIV) lag behind younger children and adults in the achievement of HIV care and treatment targets for HIV epidemic control. Treatment outcomes for adolescents may be influenced by their experiences with the support provided in HIV programs. We report on the experiences of virally unsuppressed adolescents and their caregivers with the current support in primary healthcare settings in Namibia.

**Methods:**

A qualitative descriptive and exploratory study was conducted in 13 public primary healthcare facilities in Windhoek, Namibia. A total of 25 in-depth interviews were conducted with unsuppressed adolescents (*n* = 14) and their caregivers (*n* = 11) between August and September 2023. The audio-recorded interviews were transcribed verbatim, and uploaded into ATLAS.ti software, and subjected to thematic content analysis.

**Findings:**

Three main support domains for the unsuppressed adolescents emerged from our analysis, namely: psychosocial, clinical and care, and socioeconomic support. The psychosocial support was delivered through peer support (teen clubs and treatment supporters) and enhanced adherence counselling mostly. The clinical and care support included implementing adolescent-friendly HIV services, differentiated service delivery approaches, and caregivers and healthcare worker care support for improved ART adherence, clinic attendance and continuous engagement in care. Socioeconomic support was provided for nutritional support, transport to access clinics, and school supplies, as well as income-generating projects.

**Conclusion:**

Psychosocial, clinical and care, and socioeconomic support are key elements in addressing the needs of adolescents challenged with achieving viral suppression. Health systems may benefit from whole-of-society and whole-of-government approaches to meet the needs of ALHIV that are beyond the scope of health service delivery such as nutritional, education and socioeconomic influences on both the health and well-being of ALHIV.

## 1 Introduction

Adolescents living with HIV (ALHIV) lag behind younger children and adults in the attainment of the Joint United Nations Program on HIV/AIDS (UNAIDS) and World Health Organization (WHO) targets for HIV epidemic control. Approximately 1.65 million [1.18 million-2.19 million] adolescents between the ages of 10 to 19 years were living with HIV globally in 2022 (1). Substantial resources have been committed through multi-national and country programs to reach the 95-95-95 targets (2). However, reaching these goals remains elusive, especially in children, adolescents and young adults living with HIV. Worldwide, treatment coverage lags for children and adolescents, and by 2022 only 63% [49–86%] of children living with HIV knew their HIV status, 57% [44–78%] of them were on ART, and only 46% [36–63%] were virally suppressed (81% of children on ART) (3).

An estimated 27,000 AIDS-related deaths (4% global AIDS-related mortality) were reported globally in 2022, with more than 80% of them in sub-Saharan Africa (1). Viral non-suppression is a risk factor for mortality (4), and a recent systematic review reported ART adherence levels and viral suppression for ALHIV in sub-Saharan Africa at 65 and 55%, respectively (5). An estimated 11,057 adolescents are living with HIV in Namibia, with a prevalence of 1.9% among the 10–14 years age group and 3.7% among older adolescents aged 15–19 years (6). The Namibia Population-based HIV Impact Assessment (NAMPHIA) of 2019 reported the highest annual HIV incidence among adolescents and young women aged 15–24 years (0.99% vs. 0.36% for all adults aged 15–64 years) (6). Adolescents disproportionately lag behind the adults, with a recent analysis of treatment outcomes in ALHIV in Windhoek reporting a 12 and 15% gap in viral suppression and retention in care, respectively, whilst the viral suppression gap in adults is less than 5% (7, 8).

The lower viral suppression rates in adolescents compared to adults can be attributed to the unique challenges that adolescence presents in addition to being HIV positive (9). These challenges are related to adolescence as a developmental stage that is associated with rapid physiological, psychological and physical changes, fluctuating emotions and boundary-testing behaviour (9). Barriers to achieving viral suppression include non-disclosure-related issues, stigma, lack of psychosocial support, reliance on caregivers, childhood forgetfulness, unfavourable school schedule, unavailability of transport money, medication stockouts, side effects, prolonged clinic waiting time, unfriendly health settings and socioeconomic challenges (poverty, lack of food) (5, 9–13). Noted enablers of good adherence to ART and staying engaged in HIV care include access to adolescent-friendly services, reliable drug supply chain, good attitude from healthcare workers, financial support, and family and positive peer support among others (5, 14).

The United Nations Children’s Fund (UNICEF), WHO, UNAIDS and other international partners have launched several global initiatives to end AIDS by 2030 (15), and provided some recommendations for the provision of adolescent-friendly HIV services and best practices on peer-led interventions for adolescents in HIV care. Namibia has adopted several of the international recommendations for addressing the gaps in managing ALHIV including the “*Global Plan towards ending new HIV infections among children by 2015 and keeping their mothers alive,”* and the “*2016 Political Declaration on HIV and AIDS goals”* (16, 17). Some of the most notable interventions included in the national HIV response are adolescent-friendly HIV services, differentiated care models, multi-month dispensing (MMD), peer-led interventions such as the Namibia Adolescents Treatment Supporters (NATS) and teen clubs, enhanced adherence counselling (EAC), and optimization of treatment with introduction of dolutegravir (DTG)-based regimens, as recommended by UNICEF, WHO and UNAIDS (18, 19). Despite the successes observed over the past decade to reach the 90-90-90 goals set by UNAIDS for 2020, a persistent gap remains to reach the new target of 95% viral suppression for adolescents on HIV treatment.

In 2017, Namibia attained 86-96-91% of the initial 90-90-90 UNAIDS and WHO targets for epidemic control (6). However, the most recent modelled spectrum estimates for 2023 reported that Namibia has achieved approximately 95 -97% -94% of the revised 95-95-95 UNAIDS targets. Windhoek, with an estimated population of 431,000 people, has one of the highest burdens of HIV in Namibia, and a high population of ALHIV. The estimated HIV prevalence for the younger population of 15 to 24 years is 4.0%, whereas for younger adolescents aged 10–14 years, it is estimated to be at 1.7% (20). Considering the high HIV burden among adolescents in Windhoek, the city joined the joint Fast-Track Cities Initiative, which aspires to optimize HIV service delivery, including improving treatment outcomes for ALHIV (21). A recent retrospective analysis of viral suppression among ALHIV at all the Windhoek healthcare facilities (*n* = 13) reported 12% of all the 695 adolescents on ART in Windhoek to be virally unsuppressed (1,000 copies/ml threshold) and approximately 94, 90 and 85% retention in care rates at 12, 24 and 36 months, respectively (7, 8). In this study, we report on the experiences of unsuppressed ALHIV and their caregivers with the current support in primary healthcare settings, and identify remaining, persistent gaps in service delivery to ALHIV. We believe the findings can assist with the identification of potentially modifiable determinants for enhancing viral suppression among ALHIV (22).

## 2 Methods

### 2.1 Study setting

This study was conducted in Windhoek, the capital city of Namibia. Windhoek has two main referral hospitals, two middle-level health centers, and nine primary healthcare clinics. All the 13 facilities in Windhoek were included in the study.

### 2.2 Research design, sample size, and sampling

A qualitative descriptive and exploratory study was performed. Adolescent participants (10–19 years) were obtained from the high viral load registers maintained at each health facility ART clinic. For every high viral load result received (>1,000 copies/ml), the client is enrolled into Enhanced Adherence Counselling (EAC) and registered either in the adult (>15 years) high viral load register or the paediatric (<15 years) high viral load register. The Namibia Adolescent Treatment Supporters (NATS) based at ART clinics also generate lists of adolescents with high viral loads (from the high viral load registers) who need standard or enhanced care. We generated a list of potential participants using the high viral load registers and the NATS lists for enhanced care. Adolescent participants were recruited into the study during their routine follow-up visits, with their caregivers, or the caregivers were contacted in consultation with the adolescent and clinic staff. A purposive sampling approach was utilized to enrol unsuppressed adolescents and their respective caregivers into the study, considering those in EAC and enhanced NATS care, the number of younger and older adolescents, and male and female adolescents. Written informed consent was obtained from the caregivers and older adolescents aged 18 years and above whilst younger adolescents aged less than 18 years had to assent to participate with caregiver consent. A total of 14 in-depth interviews (IDIs) were conducted with unsuppressed adolescents as well as 11 of their caregivers (Table 1), guided by the data saturation concept. Through the in-depth interviews, we explored sensitive and personal themes concerning the individual experiences of adolescents on HIV treatment, and the meanings of the disease to them. We used the interviews with caregivers to triangulate the responses of the adolescents – to enhance the credibility of the findings of this qualitative study (23).

**Table 1:** Summary characteristics of unsuppressed adolescent participants and their caregivers.

	Female	Male	Total
Age group (in years)	**10–14 (*n* = 1)**	**15–19 (*n* = 6)**	**10–14 (*n* = 2)**	**15–19 (*n* = 5)**	
In school	1	3^a^	2	3^a^	**9**
Employed				2	**2**
In teen club	1	3	2	4	**10**
Caregiver interviewed					
Father		2		1	**3**
Mother		2	1	3	**6**
Aunt			1		**1**
Grandmother	1				**1**

a 2 (1 female and 1 male) adolescents are staying in the hostels in boarding school.

### 2.3 Data collection

Separate interview guides were developed for adolescents and their caregivers and administered in person by the first author. The interview guides were developed based on the research question related to the experiences of adolescents and their caregivers with the health system, driven by preliminary quantitative analyses of determinants of viral suppression and retention in care among ALHIV in Windhoek, a review of policy and guidelines for management of ALHIV in Namibia, and key informant interviews with HIV program managers and service providers. The interview guides were piloted at two of the 13 facilities to ascertain the reliability of the guides. The interviews took place between August and November 2023. Before each interview, each participant was taken through the participant information sheet and the written informed consent for participation. All 25 IDIs were conducted in person at the ART clinics, in either English or any of the local languages, and audio recorded with the participant’s consent. The recorded interviews were transcribed verbatim and translated to English (where required).

### 2.4 Data analysis

The interview transcripts were uploaded into ATLAS.ti v8 software and we performed thematic content analysis. We developed codes from the participants` responses using an inductive approach, whereby rather than using a theoretical framework, the codes and themes emerged from the transcribed data (24). The first author first reviewed 3 adolescent transcripts and 2 caregiver transcripts (21% of all transcripts) to develop the codebook. The transcripts and codebook were reviewed by the second author and inter-coder disagreements were discussed. A comprehensive codebook was created through an iterative engagement process between the two authors, emanating in agreed codes with definitions by consensus. The emerging codes, sub-themes and themes were further reviewed, refined and verified by the authors and applied to the rest of the transcripts. Data triangulation between the adolescents and caregiver codes and themes, as well as second author checks, were employed for data validation. A final matrix of themes, sub-themes and codes describing the barriers/challenges to ART adherence and enablers/facilitators of good adherence, from the perspective of unsuppressed ALHIV and their caregivers, was developed (Supplementary materials S1, S2).

### 2.5 Rigor and trustworthiness

The credibility and trustworthiness of the research were enhanced through prolonged engagement with the healthcare facilities in Windhoek. We continuously engaged with the facilities throughout the different preceding phases of the research. We initially conducted a baseline analysis of all adolescents enrolled in ART services in all the Windhoek facilities. We then conducted a policy and programmatic documents review which involved consulting the management of the MoHSS and facility-based management and staff. During the qualitative phase of the research, we engaged with managers of adolescent-focused HIV programs and the healthcare workers providing HIV services to ALHIV. The ongoing engagement throughout the preceding data collection periods, and piloting of the interview guides helped refine the quality of questions in the data collection tools for the adolescents and their caregivers, as well as the quality of information obtained from the interviews. A participatory approach, persistent observation, and prolonged engagement enhances familiarity and understanding of contexts for the researcher, and develops a sense of ownership and involvement in the outcome of the research for the participants (25). These measures add more value for fulfilling the trustworthiness criteria in terms of the credibility of the research, and ensuring dependability, confirmability, and transferability of findings (26). The researchers also familiarized themselves with key concepts related to barriers, enablers and interventions for improving viral suppression rates, by conducting a systematic review to identify effective interventions for improving viral suppression for ALHIV. The mixed methods research design of the project, which also included a policy and programs documents review, facilitated the development of an interview guide well informed by findings from the preceding phases. As already mentioned, we also employed triangulation to analyse responses from the unsuppressed adolescents and their caregivers to compare responses to reach as rich a picture of their perspectives, experiences and different dynamics, to the extent possible and to increase credibility (23). Credibility was also enhanced by meticulous identification of unsuppressed adolescents from records and confirmed by NATS as part of the sampling process and recruitment of participants.

#### 2.5.1 Ethical approval and informed consent

The ethical clearance was obtained from the University of the Western Cape Biomedical Research Ethics Committee (ref. no. BM21/5/7) and the Namibia Ministry of Health and Social Services (MHSS) Research Management Committee (ref. no. 17/3/3/FKM). The study was carried out in compliance with the Helsinki Guidelines Declaration of 1964 and its subsequent amendments. Written consent was obtained from adolescents aged 18 years and above and parents or caregivers, and assent was obtained from adolescents under 18 years, prior to participation in the study. No personal identifying information such as names, surnames or identity numbers, was collected to ensure respect for the privacy and dignity of the participants and the confidentiality of participants’ information. All data was stored on a password-protected tablet and backed up on a password-protected computer.

## 3 Findings

Table 1 outlines the key demographics of the adolescents who participated in the study and the relations with the caregiver participants who were interviewed. A total of 25 participants were interviewed: 14 unsuppressed ALHIV and 11 caregivers of unsuppressed adolescents. Nine adolescents were in school (4 of the 7 females, 5 of the 7 males) and 2 older male adolescents were employed. All the younger adolescents (10–14 years) were in school. Ten out of the 14 adolescents were in Teen Clubs (6 males and 4 females). Their caregiver participants included mothers (*n* = 6), fathers (*n* = 3), and an aunt (*n* = 1) and a grandmother (*n* = 1).

Three main adolescents’ support domains emerged from the analysis of the interview data, namely: *psychosocial*, *clinical and care*, and *socioeconomic* support (Table 2). We describe each of these themes in turn, and note the interventions that related to each domain, as sub-themes, with verbatim quotes where appropriate to illustrate authenticity of the coding process.

**Table 2:** Support domains for ALHIV with unsuppressed viral load.

Domain	Intervention
Psychosocial support	Teen Club Peer support Namibia Adolescent Treatment Supporters (NATS) Advice on avoiding alcohol Adherence counselling Motivational interviewing
Clinical and care support	Adolescent-friendly clinic services Medication delivery at home Home visits Healthcare workers support HIV/AIDS education Facilitated medication pick-ups Introduction of long-acting injectables for HIV Weekend and after-hours clinic services Flexibility in clinic visits scheduling Receive reminders Receiving follow-up calls Caregiver support for ART adherence and clinic attendance Tracing and post-tracing services
Socioeconomic support	Food and transport support Financial support for school Savings project Accessibility of healthcare facilities

### 3.1 Psychosocial support

Adolescents who have high viral loads are enrolled for enhanced adherence counselling (EAC) and motivational interviewing, receive advice on avoiding alcohol and are enrolled in the Namibia Adolescent Treatment Supporters (NATS) enhanced care. They are also invited to the teen clubs to facilitate peer support. Both the adolescents and their caregivers described the counselling received as excellent. However, there were concerns about the availability of adequate counselling services for ALHIV with disabilities.

He is a disabled person, he is deaf. I don’t know if he receives counselling because I don’t think there are counsellors who know sign language. Long back they used to tell me, and I translate to him, for now I do not know how they do it because now he comes alone – Caregiver.

The teen clubs provide a safe environment for the adolescents to share their experiences, to support each other and learn from the facilitators (clinic staff, NATS and peers). Most adolescents reported good experiences in the teen clubs, learning the basics about HIV and AIDS, dealing with stigma and discrimination, and how to cope with their HIV status and be resilient. The caregivers concurred that the teen clubs were excellent platforms for building confidence and providing emotional support to the adolescents.

Meeting my age mates with the same status as me kept me strong, it made me to be strong, I now know that I am not alone. Sharing their stories and how they take their medicine, we seriously learn a lot – ALHIV.

This group trains them very well how to have confidence and self-competency. Giving them emotional and physical support knowing there is someone there for them. I think this group is doing the most. Knowing her she always come home hyper and energetic, I think this also boosts her – Caregiver.

However, some challenges persisted with the teen clubs. Some adolescents stopped attending the teen clubs because they felt that other members did not like them or were rude to them; or the stories shared affected them emotionally. Others stopped attending because they were staying in the hostels, or their parents refused to approve their participation in teen clubs. Some indicated that the clinic stopped calling them to come for the teen club meetings whilst others have many responsibilities at home that prevent them from attending teen club meetings.

I was introduced to it [teen club] but their dates are always not good for me. It’s either I am busy or home alone or with the kids and there is no one I will leave them with – ALHIV.

The NATS were successful in closing the gaps that existed between the adolescent clients and healthcare workers. The adolescents preferred getting assistance from the NATS at the facilities because they get preferential treatment and are fast-tracked through different service delivery points. The NATS also motivated the virologically unsuppressed adolescents to take their medication as prescribed and were role models as teenagers who overcame challenges with adherence and reached viral suppression through individual resilience. In general, most adolescents reported that the clinic staff provided excellent counselling services, as did the NATS.

I didn’t want to be seen, until one of the guys who works for NATS came in the clinic, lucky enough we know each other from school. He asked why I am leaving, and I said no I am going because your people here are acting otherwise. He was like no come let me help you they are busy. Unfortunately, he travelled, and things are now hard for me – ALHIV.

### 3.2 Socioeconomic support

Provision of food, transport money and financial support for school emerged as key enablers for adolescents struggling with adherence to treatment and clinic appointments. Nutritional support was previously provided by non-governmental organizations (NGOs) but has ceased. At the time of the study responsibility for food, transport money and school funds fell to the families – which was difficult for some (or most). An initiative to have a savings project for the adolescents was initially useful in providing transport money for clinic visits. However, it seems to have faced sustainability problems as it did not last long because of accountability concerns.

I think I should always drink them [tablets] after a meal, because sometimes there is no food, and I feel dizzy, nausea, headaches and sometimes vomit them [tablets]. Sometimes I find myself not having transport money, but facility staff members can give me – ALHIV.

The government should take it seriously. Just to provide healthy foods to our children because I don’t think these foods we are feeding them are all healthy – Caregiver.

For most of the adolescents, the clinics are quite accessible as there are short distances from their homes to the healthcare facilities. However, for those who stay longer distances from the clinics, lack of transport money becomes a barrier to accessing the clinic HIV services.

The clinic is not far, because some time I can even walk to the clinic if I do not have taxi money, the facility is just near – ALHIV.

### 3.3 Clinical and care support

Clinical services support ranged from facility-based adolescent-friendly HIV services to community-based differentiated care services. Many of the participants appreciated the prioritization of adolescents in often busy and crowded facilities, with facilitated quick medication pick-ups, especially when their clinic visits occur during school times. Some facilities have adolescent corners, which provide focused attention to the adolescents. However, participants suggested that the clinics should have more flexibility in scheduling of clinic visits, especially by involving the adolescents in decision-making on convenient times for them to come for their follow-up visits. It was also suggested to have afterhours and weekend clinic visits so as to minimize disruption of the school attendance. In addition, some participants expressed the desire to have long-acting injectables, to address the ART regimen-related challenges of taking daily medication and treatment fatigue.

The solution, what I am suggesting is the clinic should be able to provide injection for each and every month for it to replace tablets. It is irritating for the child to be responsible for taking medication each and every day – Caregiver.

Healthcare worker support through education on HIV and AIDS basics, provision of reminders (such as pill boxes, wristwatches, and sending text message reminders) are key support mechanisms offered at some of the clinics. Despite their utility, these reminders are not readily available at all facilities.

I need somewhere to check time because my father who has a phone, so I check the time is not always home. He comes late from work; I just want reminders. The support I want is to be helped with a watch – ALHIV.

Many participants indicated that the healthcare workers are friendly, supportive, helpful and always kind. However, some participants indicated that sometimes the service is slow and the clinics are often crowded with long queues.

One visit I met sister [name provided]. Mind you I used to feel guilty of not taking my medication and not coming to the clinic and everything was not up to date. When I met her, she was very understanding, nice to me, and she was encouraging me, so I told her my problem and she agreed to help me, now she went to another clinic – ALHIV.

Some of the clinics have nurses and health assistants who make follow-up calls to remind the adolescents of their upcoming clinic visits or when they have missed an appointment, which triggers the activation of tracing and post-tracing services. Receiving follow-up calls is an essential measure that helps the adolescents to comply with their visit schedules. Healthcare workers play a crucial role in promoting caregiver support and involvement in the care of the adolescents whenever such intervention is needed. It was clear from the caregivers’ responses that caregiver absence in the care of most of the unsuppressed adolescents was a key barrier to good ART adherence and clinic attendance.

If I am not around everything is messed up. That undetected condition [viral suppression] used to happen when she was under my care. I always make sure he attends his appointment. I have to keep monitoring that he takes his medication on time. The problem is mostly when I am staying in the north – Caregiver.

The NATS have been helpful in delivering medication at home for adolescents who are having problems with going to their clinics. Other adolescents who are challenged with accessing their nearest health facilities also suggested that the clinic staff should have a schedule for delivering medication at home or in the community.

I wish the nurses could bring my medication at home, then I don’t have to come here. They once told us that NATS people will be taking care of that issue. Like if you tell them your situation and they will deliver our medication at our places. I want that to be implemented – ALHIV.

The community-based support would also include conducting home visits to assess the home environment and identify and address the needs of the adolescents who are struggling because of some socioeconomic or family challenges at home, as part of the comprehensive post-tracing services.

They are even approaching exams, I need someone to come in our house and see what he is doing because I have been trying to talk to him, maybe he is hiding the medication – Caregiver.

## 4 Discussion

In this study, we qualitatively explored the experiences of unsuppressed adolescents in HIV care and their caregivers with the current support in primary healthcare settings in Windhoek, Namibia. We identified the remaining, persistent gaps in service delivery to ALHIV that related to the main barriers to ART adherence namely, psychological, social, behavioural, structural, clinical care, ART regimen-related, and socioeconomic challenges. We identified enablers such as psychosocial support, individual resilience, clinical care and structural support, which serve as the basis for our recommendations for the optimization of the HIV program for ALHIV in Namibia.

Our findings suggest that there are several good practices implemented to provide psychosocial support to ALHIV and their caregivers. The results indicate that unsuppressed ALHIV are enrolled in EAC as standard practice, and receive motivational interviewing, to motivate them to adhere to treatment (27). The counselling services provided by the clinic staff and NATS were described as excellent. Studies investigating the association between EAC and viral suppression in individuals on ART have found mixed and inconclusive results (28, 29). Nonetheless, other studies found EAC and routine viral load monitoring to be potentially effective interventions to improve viral suppression levels in individuals living with HIV (30, 31). Counselling sessions include discouraging adolescents from drinking alcohol and taking other recreational drugs which may exacerbate cognitive function and mental health impairments (32). However, there are indications that the counselling services, including EAC, are insufficient to address some unique challenges that adolescents may be facing. Concerns were raised about the accessibility of the counselling services to adolescents living with disability. The healthcare providers may not be adequately equipped to provide the necessary counselling services to clients that are deaf. A study in Kenya recommended deaf-friendly HIV services that are also supplemented by peer education programs (33).

The WHO recommends the implementation of peer support programs for adolescents and young people living with HIV (AYPLHIV) aged between 10 to 24 years, and recognizes teen clubs and community adolescent treatment supporters (CATS) as some of the best practices (34). Namibia has adopted some of these best practices and counselling services are also extended to peer support interventions such as the NATS (adopted from CATS) and teen clubs. The teen club intervention model in Namibia is primarily designed to deliver psychosocial support only, and includes unsuppressed adolescents (35). Namibia has been scaling up establishment of teen clubs and promoting greater uptake of this intervention among all ALHIV who are aware of their HIV status. Ten (71%) of the unsuppressed ALHIV who participated in our study were members of a teen club and evidently appreciate the peer support and good learning experiences in such safe spaces, as observed in a study conducted in Cape Town, South Africa (36). Great potential to improve treatment outcomes for ALHIV has been observed in group-based peer support interventions such as teen clubs. An evaluation of a teen club intervention in Malawi reported that adolescents who were not exposed to teen clubs were less likely to be retained in care than those in teen clubs (aOR 0.27; 95% CI 0.16, 0.45) (37). A 2019 evaluation in Malawi of a teen club intervention reported improved ART adherence in teen clubs (38). However, no significant association has been reported between attending a teen club and viral suppression or retention in care among ALHIV in Namibia (35, 39).

Nevertheless, several country programs are scaling up the teen club intervention, albeit as a differentiated service delivery model which includes ART refills and other clinical services (38). The teen club model in Namibia which focuses on psychosocial support only may be inadequate and could benefit from incorporating ART refills and other clinical services. Attrition from the teen clubs needs to be addressed as a number of the participants in our study indicated that they stopped attending because of negative experiences with peers (attitude, relations and emotional triggers). Other barriers to attending the teen club such as parents refusing to approve participation, staying in the hostel or being busy also need to be addressed. Adding other clinical services to the teen club and prioritizing the unique needs for each adolescent (case management for unsuppressed ALHIV), may possibly improve retention in teen clubs, and consequently their treatment outcomes. A study in Kenya recommended that facilities or organizations dealing with ALHIV should consider case management interventions to address determinants influencing adolescents` resilience in HIV treatment and care programs (40).

The NATS play a crucial role in closing some of the care gaps between adolescents, caregivers and the healthcare workers. Many of the unsuppressed adolescents have been enrolled in the NATS enhanced care and indicated that the support they get from the NATS includes facilitated pill pick-ups, being fast-tracked through the clinic during visits, encouragement to adhere to their medication, and medication deliveries at home when the adolescent cannot physically go to the clinic for their appointment. Peer-led differentiated service delivery (DSD) models have been recognised as promising interventions for improving viral suppression among ALHIV (41–43). Namibia is one of the countries currently scaling up the peer-led DSD models, and plans are underway to evaluate the effectiveness of the intervention in achieving viral suppression in unsuppressed adolescents (44). However, the NATS intervention may be inadequately implemented in Namibia, as only a few of the unsuppressed adolescents indicated that they were enrolled in the program. Concerns about the reach of the program needs further interrogation as all adolescents with unsuppressed viral loads at the study sites are expected to be under NATS enhanced care.

With the emphasis on client-centred care, it has become increasingly essential for DSD models to focus healthcare services designs on patient preferences which will promote better retention in care and is amenable to lifelong care (45). Many of the adolescent and caregiver participants suggested that medication be delivered at home, either by healthcare workers or the NATS. As mentioned earlier, NATS have taken up this responsibility especially for adolescents in enhanced care challenged with accessing the clinic, but structured community based DSD approaches for this population seems to be missing.

However, there are some **clinical and care support** best practices implemented at facility level. The clinics provide adolescent friendly services, including dedicated spaces within the facilities in the form of adolescent corners. The WHO recommends implementation of adolescent-friendly health services that improve acceptability, equity, accessibility, effectiveness, and appropriateness of ART services to ALHIV (19). Our results indicate that these services enable smoother interactions with the clinic for most adolescents, with reduced clinic waiting times and adequate clinician consultation time as needed. Yet at some health facilities, often times the clinics would be crowded and very busy, such that the service would be slow. Without prioritized assistance, long clinic waiting time becomes a significant barrier for adolescents to adhere to their clinic visit appointments, especially when they occur during school hours. Both adolescents and caregivers indicated that scheduling of some of the clinic visits was disruptive of their school attendance, with 9 of the 14 adolescents still in school. Consultations with the adolescents and caregivers gives them an opportunity to participate in the decision-making process concerning conducive scheduling of their visits. There is growing evidence that adolescents can be agents of positive change to improve their treatment outcomes and they should be critically engaged in decision-making process throughout the continuum of care (46). Suggestions to implement more flexibility in scheduling clinic visits could address clashing clinic schedules with school attendance, and this could be through after school hours and weekend scheduled visits (47).

Tracing services are key to returning clients who miss their appointments back into care. Namibia has developed a tracing and post-tracing services standard operating procedure (SOP) to guide healthcare providers on mechanisms to reduce interruption in treatment. Adolescents appreciated follow-up calls from clinicians and health assistants (lay counsellors) to remind them of upcoming appointments or if they miss their clinic visit date. Poor clinic attendance consequently predicts viral non-suppression and tracing interventions should incorporate post-tracing services that recognize the adolescent-specific individual, structural, and social barriers to uninterrupted treatment engagement (48). Our observations suggest that these services may be insufficiently implemented as some of the unsuppressed adolescents had interrupted treatment for long periods and their missed appointments should have triggered tracing services.

Our results also highlighted the great interest from adolescents and their caregivers to get assistance with reminders for taking medication. As one of the observed best practices, Namibia has introduced and distributed wristwatches and pill boxes to adolescents to facilitate timely taking of medication, although the reach of the intervention has been limited to selected facilities due to inadequate resources. Anecdotally, service providers have seen improvements in adherence in adolescents that were previously struggling with taking their medication in a timely manner. However, the effectiveness of this intervention has not been empirically established yet. Studies investigating the effectiveness of reminders (mobile phone text messages) on ART adherence have reported mixed results and recommended larger studies to be conducted (36). The Namibia HIV program plans to evaluate the effectiveness of the reminders that have been distributed to ALHIV in selected facilities.

It was apparent that some adolescents rely on their caregivers to remind them to take their medication, and in the absence of the caregiver, adherence was poor. For this reason, a few caregivers suggested the introduction of long acting injectables for HIV treatment (49). Observations from our study suggest that caregiver support, in the form of reminders, psychosocial, food and transport money, is key for adolescents to take their medication on time and to attend their clinic appointments. However, caregivers also experience challenges associated with family, psychological and social needs that require social networking and financial resources support to be strengthened as a coping mechanism for most caregivers (50). For these reasons, healthcare workers at Windhoek facilities have introduced caregivers’ clubs to provide support to the caregivers of adolescents, especially those taking care of unsuppressed ALHIV. Caregiver involvement in the treatment and care of the adolescents is key and it was apparent that problems often surfaced during caregiver absence. A caregiver has the potential to be a powerful ally for their child and family centred approaches that include socioeconomic and health needs should be prioritized to improve ART adherence and resilience among ALHIV (51).

Healthcare worker support to both the caregivers and to adolescents is essential and several participants expressed their appreciation of good support they were receiving from healthcare workers regarding psychosocial wellbeing, emotional support, motivation and encouragement to stay engaged in care and taking their medication as prescribed. Evidence suggests that health education on HIV and AIDS basics is a crucial component for improving treatment literacy among adolescents (52). HIV and AIDS education is delivered through healthcare workers, NATS and as part of the teen club activities. Interventions that target treatment literacy in ALHIV and their caregivers can address immediate determinants of poor adherence and facilitate achievement of undetectable viral loads (53). The HIV and AIDS education sessions seem to be successful in improving the understanding of HIV and AIDS basics by both adolescents and their caregivers, evidenced by the acceptance of HIV status, understanding self-management and coping mechanisms, as well as great desire to achieve viral suppression.

As alluded to earlier, structured DSD models are inadequately implemented for this population and some caregivers suggested that healthcare workers should conduct home visits to support the adolescents and their families. Addressing the socioeconomic needs of the adolescents is an essential component of managing the holistic needs of adolescents, which includes their immediate home environment. Programs providing **socioeconomic support** for ALHIV and their families have been scarce and limited. Although most of the participants stayed near the clinics, transport money was a challenge for those who stayed longer distances from the clinic. Support with transport money and food previously came through NGOs whose programs have since ended. The Bantwana Initiative provided similar support for ALHIV and their caregivers through a combination of financial literacy, savings groups, and income-generating skills to improve their financial and nutritional status, as well as food security (51). Although a savings project was initiated for some of the adolescents and caregivers in Windhoek, it was not sustainable.

Because of the socioeconomic challenges raised by participants, we observed that there is a significant gap in addressing nutritional, transport, and financial support for school supplies. Designing programs specifically addressing socioeconomic challenges for ALHIV may go a long way in addressing some of the structural determinants of poor adherence and viral non-suppression. In addition, it appears support from social workers is missing or inadequate, at most, as there were no clear engagements mentioned specifically with social workers by the adolescents nor their caregivers. Overall, it is essential to involve the adolescents and their caregivers in designing programs that are concerned with their care, and utilizing the whole-of-society and whole-of-government approaches to address the holistic needs of ALHIV (54).

The limitations of our study included the smaller number of younger adolescent participants compared to the older adolescents, and our findings may be predominantly the experiences of older adolescents who are challenged with attaining and maintaining viral suppression. Our study setting was also limited to Windhoek, with facilities in urban and peri-urban settings. The experiences of adolescents in rural settings may be different. Our study focus was on unsuppressed adolescents and did not include interviews with suppressed adolescents.

## 5 Conclusion

Psychosocial, clinical and care, and socioeconomic support are key elements in addressing the needs of adolescents challenged with achieving viral suppression. We recommend that the involvement of ALHIV and their caregivers should be part of a whole-of-society approach for health service delivery. In addition, direct involvement of other sectors such as education, social welfare, and poverty eradication through a whole-of-government approach, is essential to meet the needs that are beyond the scope of health services, but directly impact the health and well-being of ALHIV such as nutritional, education and socioeconomic determinants of health. Practices of adolescent HIV care should be holistic and participatory for both ALHIV and their caregivers to achieve optimum outcomes, including sustained viral suppression.

## Data availability statement

The original contributions presented in the study are included in the article/Supplementary material, further inquiries can be directed to the corresponding author.

## Ethics statement

The studies involving humans were approved by University of the Western Cape Biomedical Research Ethics Committee. The studies were conducted in accordance with the local legislation and institutional requirements. Written informed consent for participation in this study was provided by the participants’ legal guardians/next of kin.

## Author contributions

FM: Conceptualization, Data curation, Formal analysis, Investigation, Methodology, Project administration, Resources, Software, Validation, Writing – original draft, Writing – review & editing. BW: Conceptualization, Data curation, Formal analysis, Methodology, Supervision, Validation, Writing – review & editing.
